# Méningo-encéphalite à entérobactérie multirésistante dans la zone guinéo-malienne

**DOI:** 10.48327/mtsi.v4i3.2024.452

**Published:** 2024-07-04

**Authors:** Ouo-Ouo LOUA, Issa KONATÉ, Yacouba CISSOKO, Mamadou Saliou SOW

**Affiliations:** 1 Service des maladies infectieuses et tropicales du Centre hospitalier universitaire (CHU) point G de Bamako, Mali; 2 Université des sciences, des techniques et des technologies de Bamako, Mali; 3 Service des maladies infectieuses et tropicales du Centre hospitalier universitaire (CHU) de Donka, Conakry, République de Guinée; 4 Université Gamal Abdel Nasser de Conakry, République de Guinée

**Keywords:** *Escherichia coli*, Méningo-encéphalite, Multirésistant, Antibiotiques, Sepsis, CHU Point G, Bamako, Mali, Guinée, Afrique subsaharienne, *Escherichia coli*, Meningoencephalitis, Multidrug-resistant, Antibiotics, Sepsis, Point G University Hospital, Bamako, Mali, Guinea, Sub-Saharan Africa

## Abstract

Les infections à entérobactéries sécrétrices de béta-lactamase à spectre étendu (E-BLSE) constituent un problème de santé publique. Nous rapportons ici deux cas de méningo-encéphalite à *Escherichia coli* multirésistante observés au Service des maladies infectieuses et tropicales du Centre hospitalier universitaire Point G de Bamako chez deux jeunes femmes. La première, qui avait avorté récemment, était sous traitement par ceftriaxone quand elle a été admise pour syndrome méningé et syndrome pyramidal lié à une lésion cérébrale. Du liquide céphalorachidien (LCR), du sang et d'un prélèvement de sécrétions génitales purulentes a été isolé *E. coli* résistant aux pénicillines et céphalosporines. Elle a guéri sous traitement par méropénem pendant 21 jours, avec peu de séquelles. La seconde avait accouché dans les semaines qui ont précédé la survenue d'un sepsis. Elle présentait une méningite et un syndrome pyramidal avec des lésions cérébrales. Du LCR et du prélèvement génital a été isolé *E. coli* résistant à de nombreux antibiotiques. Elle a guéri (avec séquelles) sous traitement par meropenem pendant 21 jours. Ces deux observations montrent que les infections génitales féminines *(post partum* ou *post abortum)* à *E. coli* peuvent être à l'origine de formes aussi graves que des méningo-encéphalites. Des souches productrices de E-BLSE peuvent être en cause, ce qui constitue un défi thérapeutique majeur. Le monde court un risque de pandémie incurable à bactéries hautement résistantes et émergentes (BHRe) en l'absence de réglementation sur l'usage des antibiotiques, de prévention et de contrôle des infections.

## Abstract (en)

Extended-spectrum beta-lactamase-secreting Enterobacteriaceae (ESBL-E) infections constitute a real global public health problem, particularly in sub-Saharan Africa. We report here two cases of ESBL-E meningoencephalitis in young female patients occurring in a gestational context observed at the infectious and tropical diseases department of the Point G university hospital in Bamako. The first woman, who recently had an abortion, was being treated with ceftriaxone when she was admitted for meningeal syndrome and pyramidal syndrome associated with brain damage. *Escherichia coli* resistant to penicillins and cephalosporins was isolated from cerebrospinal fluid (CSF), blood and a purulent genital swab. She recovered under meropenem treatment for 21 days, with few sequelae.

The second had given birth in the weeks prior to the onset of generalized infection. She was admitted for meningeal syndrome and pyramidal syndrome associated with several cerebral lesions disclosed by the CT scan. Multidrug-resistant *E. coli* was isolated from CSF and genital purulent sample. She recovered (with sequelae) under treatment with meropenem for 21 days. These two observations show that female genital infections *(postpartum* or *post abortum)* with *E. coli* can cause forms as serious as meningoencephalitis, in addition to the abortion they cause. ESBL-E -producing strains may be involved and represent a major therapeutic challenge. Better infection prevention and control would prevent multi-drug-resistant infections and their spread. Regulating the use of carbapenems and other antibiotics still effective against multi-resistant bacterial strains within the framework of global health (one health) would avert an incurable pandemic of highly resistant and emerging bacteria (HRB).

## Introduction

Les infections à entérobactéries multirésistantes constituent un réel problème de santé publique en Afrique sub-saharienne [2, 10, 17]. *Escherichia coli* est une bactérie de la flore intestinale humaine normale [13]. C'est aussi l'agent de nombreuses infections communautaires (urinaires, génitales, digestives) ou nosocomiales (ex. infection du site opératoire), dues souvent à des souches résistantes à de nombreux antibiotiques. Les bactériémies sont fréquentes. Les méningites ont la réputation d’être rares et d’être rencontrées essentiellement chez les enfants de moins de 5 ans (méningites néonatales surtout) et chez les immunodéprimés [1, 2, 8, 14, 9, 10, 13, 18].

Nous rapportons ici deux cas de méningo-encéphalite à *E. coli* multirésistant au service des maladies infectieuses et tropicales du Centre hospitalier universitaire Point G de Bamako.

## 1^er^ cas clinique

Patiente de 32 ans, cultivatrice, en provenance de la Guinée, admise dans le service le 8 juin 2022 pour fièvre et conscience altérée. Sa symptomatologie serait d'installation progressive depuis dix jours environ, précédée d'une fièvre permanente, de céphalées et de vomissements. Elle est apparue après une révision utérine dans une structure sanitaire guinéenne le 25 mai 2022 pour avortement spontané d'une grossesse de 18 semaines d'aménorrhée. Elle était traitée avant son admission pour méningite bactérienne et infection génitale suspecte par ceftriaxone et métronidazole injectables. Elle n'avait aucun antécédent médico-chirurgical connu, ni de notion d'usage d'immunosuppresseurs. L'examen physique de la patiente a objectivé un indice de masse corporelle (IMC) à 20,9 kg/m^2^ pour un poids de 61 kg, une fièvre à 39,2°C, un score SOFA *(Sequential Organ Failure Assessment)* à 3 (1 pour la pression artérielle moyenne à 65 mmHg et 2 pour le score de Glasgow à 11/15), des convulsions tonicocloniques généralisées, une raideur méningée, une hémiplégie droite. Il existait une infection génitale : lochies purulentes, utérus pelvien non retracté.

**Figure 1:**
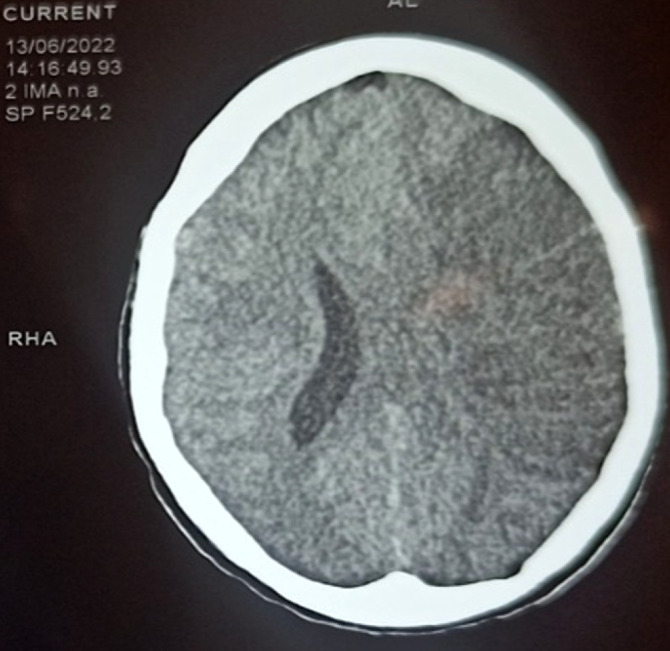
Tomodensitométrie (TDM) crânio-cérébrale avant injection du produit de contraste : large plage d'hypodensité hémisphérique gauche

**Figure 2:**
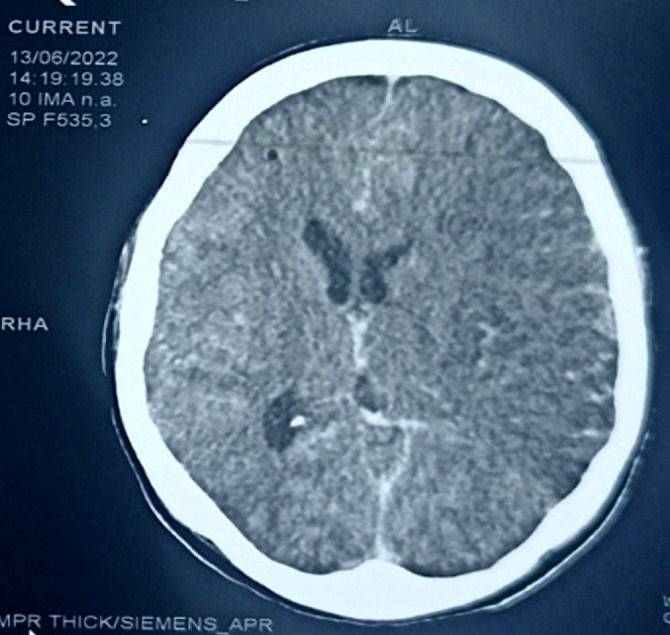
TDM crânio-cérébrale après injection de produit de contraste : rehaussement gyriforme en doigt de gant de la large plage d'hypodensité hémisphérique gauche, avec un effet de masse sur le ventricule latéral homolatéral collabé ainsi que la faux du cerveau qui est déviée à droite de 1 cm, et un effacement des sillons en regard de la lésion

La tomodensitométrie crânio-cérébrale a mis en évidence une large plage d'hypodensité hémisphérique gauche (Fig. 1), rehaussée par le produit de contraste avec important effet de masse (Fig. 2). Au bilan biologique, on constatait une hypoglycémie capillaire à 3,2 mmol/l, une clairance de la créatinine (selon la formule CKDEPI) à 155,32 ml/mn pour une créatininémie à 38,5 µmol/l, des transaminases ALAT à 58 Ul/l, et un groupe sanguin O positif. L'examen du liquide cérébro-spinal (LCS) montrait un liquide purulent, une hyperleucocytorachie à 1 100/mm^3^ à prédominance neutrophile, une hypoglycorachie à 1,2 mmol/l, une hyperprotéinorachie à 1,8 g/l, des bacilles gram négatif *E. coli* résistants aux pénicillines (amoxicilline, ampicilline, amoxiacide clavulanique), aux carboxypénicillines (ticarcilline), aux céphalosporines (ceftriaxone, cefixime, cefalotine, cefepime), aux quinolones (ciprofloxacine, norfloxacine), aux cyclines (tétracycline) et au cotrimoxazole.

L'hémoculture sur milieu aérobie et l'examen du prélèvement vaginal ont isolé la même souche. L'hémogramme a mis en évidence une anémie sévère (à 4,2 g/dl d'hémoglobine), normocytaire (VGM à 85,2 fl), normochrome (CCMH à 32,7 g/dl), régénérative (réticulocytes à 160 g/l), des leucocytes à 8 000/mm^3^ (neutrophiles à 6 200/mm^3^, lymphocytes à 1 000/mm^3^) et des plaquettes à 14 000/µl. Deux sérologies VIH se sont révélées négatives, et le taux de CD4 était à 850 cellules/mm^3^ de sang. Le diagnostic de sepsis *post abortum,* compliqué de méningoencéphalite à *E. coli* multirésistant et d'anémie sévère a alors été retenu.

Dès l'admission, elle avait été mise sous amoxiacide-clavulanique en raison de 2 g toutes les huit heures et amikacine 1 g/jour en intraveineuse. Au 4^e^ jour, à la suite des résultats de l'antibiogramme, le traitement par méropenem a été instauré à raison de 2 g toutes les huit heures en intraveineuse pendant 21 jours. L'anémie sévère a été corrigée par la transfusion journalière de 450 ml de concentré érythrocytaire isogroupe-isorhésus pendant six jours. L'hypoglycémie a été corrigée par une perfusion de 500 ml de sérum glucosé 10 %, les convulsions par l'administration de diazépam. Par ailleurs, des toilettes génitales biquotidiennes à la chlorhexidine pendant cinq jours et de la kinésithérapie au 8^e^ jour du traitement par méropenem ont été réalisées. L’évolution a été favorable, marquée par l'apyrexie au 4^e^ jour de la bi-antibiothérapie, l'amendement des convulsions, et la reprise de la conscience respectivement au 2^e^ et au 7^e^ jour du traitement par méropenem. L'hémoglobine de contrôle est revenue à 11,9 g/ dl au 7^e^ jour de la transfusion sanguine. La force motrice des membres droits déficitaires a été mesurée à 1/5 et 3/5 respectivement au 7^e^ et au 4^e^ jour de la kinésithérapie. L'exéat a été autorisé le 5 juillet 2022, avec kinésithérapie en ambulatoire et un rendez-vous quatorze jours après. À ce rendez-vous, l’évolution était globalement favorable avec une force motrice des membres initialement déficitaires à 5/5.

## 2^e^ cas clinique

**Figure 3:**
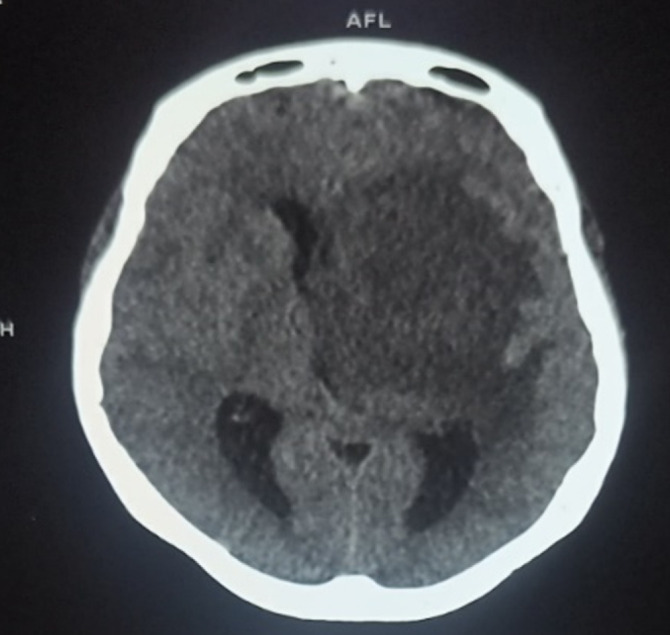
TDM crânio-cérébrale avant l'injection du produit de contraste, mettant en évidence des foyers d'hypodensité dont un occupant la quasi-totalité de l'hémisphérique gauche avec effet de masse sur le ventricule homonyme et déviant la faux du cerveau à droite, et l'autre périventriculaire droite

**Figure 4:**
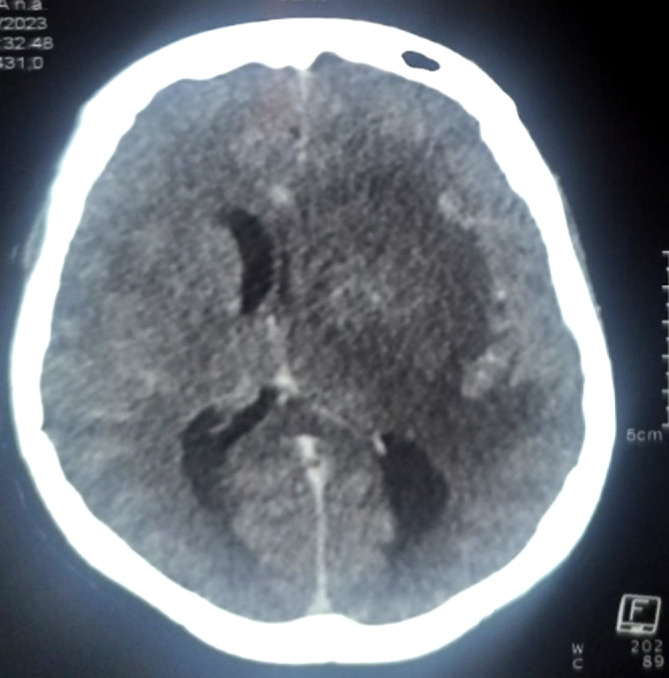
TDM crânio-cérébrale après injection du produit de contraste de la seconde patiente de 29 ans atteinte de méningo-encéphalite à E-BLSE, mettant en évidence un rehaussement des foyers d'hypodensité décrits dans la Figure 3

Patiente de 29 ans, ménagère, résidant à Bamako, admise dans le service le 20 mars 2023 pour conscience altérée et fièvre. Sa symptomatologie serait d'installation progressive évoluant depuis environ quatorze jours, marquée par de la fièvre, des céphalées et vomissements, puis par une impotence fonctionnelle des membres droits. Elle avait été traitée avant son admission pour paludisme grave confirmé, à base d'artésunate en injection dans une structure sanitaire privée durant 6 jours. Elle avait accouché vers le 14 février 2023 après avoir suivi correctement les consultations prénatales. Son enfant serait bien portant. Elle n'avait aucun antécédent médicochirurgical connu, ni de notion d'usage d'immunosuppresseurs. L'examen physique a objectivé une fièvre à 38,8°C, un score SOFA à 6 (3 pour le score de Glasgow à 9/15 et 3 pour une diurèse journalière à 490 ml), une raideur de la nuque, un syndrome pyramidal (hémiplégie et aréflexie droite, hémiparésie gauche avec force motrice à 4/5 au membre supérieur et 3/5 au membre inférieur), et les doigtiers du toucher vaginal souillés de pertes abondantes. La tomodensitométrie crânio-cérébrale a mis en évidence plusieurs foyers hypodenses (Fig. 3) rehaussés par le produit de contraste (Fig. 4). L'un de ces foyers occupait la quasi-totalité de l'hémisphère gauche, avec effet de masse sur le ventricule homonyme et déviant la faux du cerveau à droite, l'autre foyer étant périventriculaire droit. L'examen du LCS montrait un liquide trouble, une hyperleucocytorachie panachée à 1 080/mm^3^, une hypoglycorachie à 0,5 g/l et une hyperprotéinorachie à 1,2 g/l. Du LCS et du prélèvement vaginal ont été isolées des souches *d'E. coli* résistantes aux pénicillines (amoxicilline, amoxi-acide clavulanique), aux carboxypénicillines (ticarcilline), à un aminoside (gentamycine), aux céphalosporines (ceftriaxone, cefixime, cefalotine, cefepime), aux quinolones (ciprofloxacine, norfloxacine), à une cycline (tétracycline), aux phénicolés (chloramphénicol) et au cotrimoxazole. Elles étaient sensibles au meropenem, à l'amikacine, la ceftazidine, la colistine et la cefoxitine. La clairance de la créatinine (selon la formule CKD-EPI) était à 61,35 ml/mn pour une créatininémie à 110 µmol/l. L'hémogramme a mis en évidence une neutropénie modérée à 1 400/ml et une anémie modérée (à 9,1 g/dl d'hémoglobine), microcytaire (VGM à 79 fl), hypochrome (CCMH à 30 g/dl). Deux sérologies VIH se sont avérées négatives. Le diagnostic de sepsis à porte d'entrée génitale, compliqué de méningo-encéphalite suppurée à *E. coli* multirésistant et d'insuffisance rénale aiguë a été retenu.

La patiente a alors été mise sous méropenem à raison de 1 g toutes les huit heures en intraveineuse pendant 21 jours. Elle a par ailleurs bénéficié d'un apport hydro-électro-calorique à raison de 3 litres par jour, de toilettes génitales biquotidiennes à la chlorhexidine pendant cinq jours, puis de kinésithérapie dès le 10^e^ jour du traitement par méropenem. L’évolution a été favorable, marquée par l'apyrexie et la reprise de la conscience respectivement au 3^e^ et au 6^e^ jour du traitement par méropenem; la force motrice des membres a été évaluée à 2/5 à droite et 5/5 pour le membre supérieur gauche, et à 4/5 pour le membre inférieur gauche au 12^e^ jour de la kinésithérapie; la fonction rénale a été bonne au 10^e^ jour du traitement par méropenem. L'exéat a été autorisé le 14 avril 2023, avec des séances de kinésithérapie en ambulatoire et un rendezvous dix jours après. À ce rendez-vous, la force motrice des membres a été évaluée à 5/5 à gauche et 3/5 à droite.

## Discussion

Les méningo-encéphalites à *E. coli* surviennent généralement chez les enfants de moins de 5 ans notamment en période néonatale. On observe avec une fréquence accrue des sepsis avec localisation méningée chez les adultes immunodéprimés (patients infectés par le VIH, leucémiques, neutropéniques, ou sous thérapies immunosuppressives [5, 6, 18]). Le sepsis constitue dans la majorité des cas une infection liée aux soins [1, 4, 8]. L'atteinte cérébro-méningée à *E. coli* multirésistant reste rare dans la littérature; un abcès cérébral peut se développer après une phase pré suppurative (cérébrite), comme chez la première patiente.

La relation avec une infection génitale par cette entérobactérie retrouvée chez nos patientes est fréquente dans la littérature [3, 20]. La dissémination hématogène de la bactérie à l'occasion d'une endométrite à *E. coli post partum* ou *post abortum* peut être liée à une infection communautaire [20], ou à la suite de son inoculation génitale iatrogène lors de la révision utérine comme observé au cours d'autres gestes invasifs [1, 4, 8].

Les arguments diagnostiques du sepsis et de la méningite retrouvés chez nos patientes sont décrits dans la littérature [18].

Le profil de résistance de cette bactérie est similaire à celui observé dans plusieurs études [1, 7, 10, 11, 16, 20] : ces souches correspondent phénotypiquement aux entérobactéries sécrétrices de béta-lactamase à spectre étendu (E-BLSE) rapportées dans la littérature [16]. La fréquence de ces infections à entérobactéries multirésistantes est due à l'usage généralisé et souvent inapproprié des antibiotiques auxquels elles étaient naturellement sensibles. La dissémination hématogène de ces entérobactéries et le franchissement de la barrière hématoméningée chez les patientes dont nous relatons l'observation s'expliqueraient par l'immunodépression relative liée à la grossesse, la neutropénie et/ou l'insuffisance rénale. Les méningo-encéphalites sur terrain gestationnel signalées le plus souvent dans la littérature sont liées à *Listeria monocytogenes,* dont les sepsis sont favorisés par certaines immunodéficiences [12, 15]. Ces deux cas cliniques prouvent que *E. coli* peut aussi être impliqué dans des sepsis allant jusqu’à la survenue de méningo-encéphalites sur ces terrains. Devant un tableau de méningite ou de méningo-encéphalite dans les suites en *post partum* ou *post-abortum,* surtout s'il existe une endométrite associée, la notion d'une révision utérine, d'une antibiothérapie récente par béta-lactamines, il est justifié de proposer un traitement empirique d'urgence avec des antibiotiques efficaces sur *E. coli* multirésistant et *L. monocytogenes.* Le méropenem est la solution qui a réussi chez nos deux patientes, mais, compte tenu de la notion des souches de *L. monocytogenes* résistantes aux carbapénèmes [19], on pourrait envisager une antibiothérapie empirique comportant en plus l'amoxicilline et l'amikacine.

## Conclusion

Les infections à E-BLSE communautaires et liées aux soins deviennent récurrentes en Afrique sub-saharienne. Les formes les plus graves sont le sepsis et la méningite ou la méningo-encéphalite. La multirésistance de certaines souches bactériennes comme celles d'E. *coli* interpelle sur le traitement empirique des infections (même communautaires) qu'il faudrait réviser, et sur la bonne prévention et le contrôle des infections. L'usage des carbapénèmes et autres antibiotiques encore efficaces contre les souches bactériennes multirésistantes doit être réglementé. À défaut, leur usage inapproprié serait à l'origine d'une pandémie incurable à bactéries hautement résistantes et émergentes (BHRe).

Le risque d’émergence de souches résistantes est souvent lié aux activités de soins : les praticiens doivent observer les règles d'asepsie rigoureuse, et veiller au bon usage des antimicrobiens. La résistance aux antimicrobiens (RAM) est une notion qui doit être particulièrement inculquée dans les écoles de médecine au même titre que les maladies à potentiel épidémique. L'utilisation d'antibiotiques en automédication doit être évitée.

## Consentement éclairé

Nos patientes ont consenti à la publication de leurs dossiers médicaux sous anonymat.

## Remerciements

Nos remerciements s'adressent aux docteurs Mariame Soumaré, Dramane Sogoba, et Oumar Magassouba, ainsi qu'au professeur Sounkalo Dao, pour leurs contributions dans la prise en charge des patientes et pour leurs apports critiques à cet article.

## Contribution des auteurs

Ouo-Ouo LOUA : Conception du cas clinique, prise en charge de la patiente, revue de littérature, rédaction du manuscrit.

Issa KONATÉ et Yacouba CISSOKO : Prise en charge des patientes, apport critique et correction de la version finale à publier.

Mamadou Saliou SOW : Apport critique et correction de la version finale à publier.

## Conflicts of interest

The authors declare no conflict of interest.
