# Supplementary material for: Multidrug-resistant Enterobacteriaceae meningoencephalitis in the Guinean-Malian area
Source: Med Trop Sante Int. 2024 Jul 4;4(3):mtsi.v4i3.2024.452. [Article in French] doi: 10.48327/mtsi.v4i3.2024.452 (PMC11809063; doi:10.48327/mtsi.v4i3.2024.452)
Supplement: Related texts

# Research article: Multidrug-resistant Enterobacteriaceae meningoencephalitis in the Guinean-Malian area

## Introduction

Multi-resistant enterobacterial infections are a serious public health problem in sub-Saharan Africa [2, 10, 17]. *Escherichia coli* is a bacterium found in the normal human intestinal flora [13]. It also can cause numerous community (urinary, genital and digestive) and nosocomial infections (e.g. surgical site infections). These are often due to strains resistant to many antibiotics. Bacteremia is common. Meningitis is rare, mostly affecting children under 5 years of age (especially neonatal meningitis) and in immunocompromised patients [1, 2, 8, 14, 9, 10, 13, 18].

We report two cases of multi-resistant *E. coli* meningoencephalitis in the infectious and tropical diseases department of the Point G University Hospital in Bamako.

## 1st clinical case

The patient, a 32-year-old farmer from Guinea, was admitted to the department on 8 June 2022 with fever and altered consciousness. Her symptoms had been progressive for about 10 days, preceded by persistent fever, headache and vomiting. The fever began on 25 May 2022 following a uterine revision at a Guinean health facility for spontaneous abortion of an 18-week amenorrhea pregnancy. Prior to admission, she had been treated with injectable ceftriaxone and metronidazole for bacterial meningitis and genital infection. She had no known medical or surgical history and no history of immunosuppressive drug use. A physical examination revealed a body mass index (BMI) of 20.9 kg/m2 for a weight of 61 kg and fever of 39.2°C, Sequential Organ Failure Assessment (SOFA) score of 3 (1 for mean arterial pressure of 65 mmHg and 2 for Glasgow score of 11/15), generalized tonic-clonic convulsions, meningeal stiffness and right hemiplegia. There was a genital infection: purulent lochia, pelvic uterus not retracted.

A craniocerebral computed tomography scan revealed a large area of hypodensity in the left hemisphere (Fig. 1), enhanced by contrast medium with a significant mass effect (Fig 2). Biological findings included capillary hypoglycemia of 3.2 mmol/L, creatinine clearance (using the CKD-EPI formula) of 155 ml/min at a creatinine level of 38.5 µmol/L, ALAT transaminases of 58 IU/L and blood group O positive. Cerebrospinal fluid (CSF) examination showed purulent fluid, hyperleukocytorachia at 1100/mm3 with a predominance of neutrophils, hypoglycorachia at 1.2 mmol/L, hyperproteinorachia at 1. 8 g/L, gram-negative *E. coli* bacilli resistant to penicillins (amoxicillin, ampicillin, amoxiclavulanic acid), carboxypenicillins (ticarcillin), cephalosporins (ceftriaxone, cefixime, cefalotin, cefepime), quinolones (ciprofloxacin, norfloxacin), cyclins (tetracycline) and cotrimoxazole.

**Figure 1:** Craniocerebral CT before injection of the contrast product: wide range of left hemispheric hypodensity

**Figure 2:** Craniocerebral CT after injection of contrast product: gyriform enhancement like a thimble of the wide range of left hemispheric hypodensity, with a mass effect on the collapsed ipsilateral lateral ventricle as well as the falx cerebri which is deviated to the right by 1 cm, and an erasure of the furrows next to the lesion

Blood culture on aerobic medium and examination of the vaginal swab isolated the same strain. The hemogram showed severe anemia (hemoglobin 4.2 g/dL), normocytic (GMV 85.2 fl), normochromic (HMCF 32.7 g/dL), regenerative (reticulocytes 160 g/L), leukocytes 8,000/mm3 (neutrophils 6,200/mm3, lymphocytes 1,000/mm3) and platelets 14,000/µL. Two HIV serologies were negative and the CD4 count was 850 cells/mm3 of blood. The diagnosis of post-abortal sepsis complicated by multi-resistant *E. coli* meningoencephalitis and severe anemia was then adopted. On admission, she was started on amoxicillin-clavulanic acid 2 g every eight hours and amikacin 1 g/day intravenously. On the 4th day, following the results of the antibiogram, treatment with meropenem was started at 2 g every eight hours intravenously for 21 days. Severe anemia was corrected by daily transfusion of 450 mL type-specific packed erythrocytes for six days. Hypoglycemia was corrected by an infusion of 500 mL of 10% glucose serum, and convulsions were corrected by the administration of diazepam. In addition, genital shower with chlorhexidine twice a day for five days and physiotherapy on the 8th day of treatment with meropenem were performed. The course was favorable, with apyrexia on the 4th day of dual antibiotic treatment, improvement in convulsions and regaining consciousness on respectively the 2nd and 7th days of meropenem treatment. The hemoglobin returned to 11.9 *g/dL* on the 7th day of blood transfusion. Motor strength in the affected right limbs was measured at 1/5 and 3/5 on the 7th and 14th days of physiotherapy, respectively. The discharge was approved on 5 July 2022, with outpatient physiotherapy and a follow-up appointment 14 days later. At this appointment, overall progress was favorable, with motor strength in the initially impaired limbs at 5/5.

## 2nd clinical case

The patient, aged 29, housewife, resident in Bamako, was admitted to the service on 20 March 2023 with altered consciousness and fever. Her symptoms were progressive and had been developing for about 14 days, characterized by fever, headache and vomiting, followed by functional impotence of the right limbs. Prior to admission, she had been treated with artesunate injections for six days at a private health facility for confirmed severe malaria. She gave birth on or around 14 February 2023 after receiving adequate antenatal care. Her baby was reported to be in good health. She had no known medical or surgical history and was not on any immunosuppressive medication. Physical examination revealed a fever of 38.8°C, SOFA score of 6 (3 for Glasgow score of 9/15 and 3 for daily diuresis of 490 mL), stiff neck, pyramidal syndrome (right hemiplegia and areflexia, left hemiparesis with motor strength of 4/5 in the upper limb and 3/5 in the lower limb). Vaginal touch fingertips were stained with copious discharge. A craniocerebral computed tomography scan showed several hypodense foci (Fig. 3), which were enhanced by contrast (Fig. 4). One of these foci occupied almost the entire left hemisphere, with mass effect on the homonymous ventricle and rightward deviation of the falx cerebri; the other focus was periventricular on the right. CSF examination revealed a turbid fluid, hyperleukocytorachia of 1080/mm3, hypoglycorachia of 0.5 g/L and hyperproteinorachia of 1.2 g/L. *E. coli* strains resistant to penicillins (amoxicillin, amoxiclavulanic acid), carboxypenicillins (ticarcillin), an aminoglycoside (gentamycin), cephalosporins (ceftriaxone, cefixime, cefalotin, cefepime), quinolones (ciprofloxacin, norfloxacin), a cyclin (tetracycline), phenicols (chloramphenicol) and cotrimoxazole were isolated from the CSF and vaginal swab. They were sensitive to meropenem, amikacin, ceftazidine, colistin and cefoxitin. Creatinine clearance (using the CKD-EPI formula) was 61.35 ml/min with a blood creatinine level of 110 µmol/L. The hemogram showed moderate neutropenia at 1,400/mL and moderate anemia (hemoglobin 9.1 g/dL), microcytic (GMV 79 fl), hypochromic (HDAC 30 g/dL).

**Figure 3:** Cranio-cerebral CT before the injection of the contrast product highlighting foci of hypodensity including one occupying almost the entirety of the left hemispheric with mass effect on the homonymous ventricle and deflecting the falx of the brain to the right, and the other right periventricular

**Figure 4:** Cranio-cerebral CT after injection of the contrast product of the second 29-year-old patient suffering from ESBL-E meningoencephalitis, highlighting an enhancement of the hypodensity foci described in Figure 3

Two HIV serologies were negative. The diagnosis of sepsis with genital route of entry complicated by purulent meningoencephalitis caused by multi-resistant *E. coli* and acute renal failure was accepted.

The patient was then started on meropenem 1 g intravenously every eight hours for 21 days. She was also given 3 liters of fluid and electrolytes per day, and twice-daily genital shower with chlorhexidine for five days, followed by physiotherapy from the tenth day of meropenem treatment. Progress was favorable, with apyrexia and return of consciousness on the 3rd and 6th days of meropenem treatment respectively; limb motor strength was assessed at 2/5 on the right and 5/5 for the left upper limb, and 4/5 for the left lower limb on the twelfth day of physiotherapy; renal function was good on the 10th day of meropenem treatment. The discharge was authorized on 14 April 2023, with outpatient physiotherapy sessions and an appointment 10 days later. At this appointment, the strength of the left and right limbs was assessed as 5/5 and 3/5, respectively.

## Discussion

*E. coli* meningoencephalitis usually occur in children under 5 years of age, especially in the neonatal period. Immunocompromised adults (patients with HIV infection, leukemia, neutropenia or undergoing immunosuppressive therapies [5, 6, 18]) are more prone to meningeal sepsis. In the majority of cases, sepsis is a healthcare-associated infection [1, 4, 8]. Cerebral-meningeal involvement by multi-resistant *E. coli* remains rare in the literature; a cerebral abscess may develop after a pre-suppurative phase (cerebritis), as in the first patient.

The association with genital infection with this enterobacterium found in our patients has been frequently reported in the literature [3, 20]. The hematogenous spread of the bacterium in postpartum or postabortion *E. coli* endometritis may be related to a community infection [20] or to iatrogenic genital inoculation during uterine revision, as observed in other invasive procedures [1, 4, 8]. The diagnostic arguments for sepsis and meningitis found in our patients are described in the literature [18].

The resistance profile of this bacterium is similar to that observed in several studies [1, 7, 10, 11, 16, 20]: these strains correspond phenotypically to the extended-spectrum beta-lactamase-secreting Enterobacteriaceae (E-BLSE) reported in the literature [16]. The frequency of these multi-resistant enterobacteria infections is due to the widespread and often inappropriate use of antibiotics. The hematogenous dissemination of these enterobacteria and the crossing of the hematomeningeal barrier in the patients reported here can be explained by the relative immunosuppression associated with pregnancy, neutropenia and/or renal failure. The meningoencephalitis in pregnancy most frequently reported in the literature is associated with *Listeria monocytogenes,* whose sepsis is favored by certain immunodeficiencies [12, 15]. These two clinical cases demonstrate that *E. coli* can also be involved in sepsis, even leading to meningoencephalitis. In the event of meningitis or meningoencephalitis in the post-partum or post-abortal period, especially if there is an associated endometritis, a history of uterine revision, or recent antibiotic treatment with beta-lactam antibiotics, it is advisable to offer empirical emergency treatment with antibiotics that are effective against multi-resistant *E. coli* and *L. monocytogenes.* Meropenem proved to be an efficacious treatment in our two patients. However, given the prevalence of carbapenem-resistant strains of *L. monocytogenes* [19], it may be possible to consider empiric antibiotic therapy in addition to amoxicillin and amikacin.

## Conclusion

Community-acquired and healthcare-associated E-BLSE infections are becoming increasingly prevalent in sub-Saharan Africa. The most severe manifestations are sepsis and meningitis or meningoencephalitis. The emergence of multi-resistant bacterial strains, such as *E. coli,* necessitates a reassessment of empirical infection treatment regimens, even in community-acquired cases, and a renewed focus on infection prevention and control measures. The utilization of carbapenems and other antibiotics that remain efficacious against multi-resistant bacterial strains necessitates regulatory oversight. Otherwise, the inappropriate use of these antibiotics could result in the emergence of a highly resistant and emerging bacteria (HREB) incurable pandemia. The risk of emergence of resistant strains is often linked to healthcare activities. It is therefore imperative that practitioners observe rigorous asepsis rules and ensure the correct use of antimicrobials. It is imperative that antimicrobial resistance (AMR) be included in the curriculum of medical schools, in the same manner as diseases with epidemic potential. Self-medication with antibiotics must be avoided.

## Informed consent

Our patients consented to the publication of their medical records anonymously.

## Acknowledgments

We would like to thank Dr Mariame Soumaré, Dr Dramane Sogoba, Dr Oumar Magassouba and Professor Sounkalo Dao for their contributions to the care of the patients and their critical contributions to this article.

## Authors’ contributions

Ouo-Ouo LOUA: Design of the clinical case, patient management, literature review, drafting of the manuscript.

Issa KONATÉ and Yacouba CISSOKO: Patient management, critical input and correction of the final version to be published.

Mamadou Saliou SOW: Critical input and correction of the final version to be published.

## Conflits d'intérêts

Les auteurs ne déclarent aucun conflit d'intérêt.
